# Peptide Toxin Diversity and a Novel Antimicrobial Peptide from the Spider *Oxyopes forcipiformis*

**DOI:** 10.3390/toxins16110466

**Published:** 2024-10-31

**Authors:** Kexin Wang, James Mwangi, Kaixun Cao, Yi Wang, Jinai Gao, Min Yang, Brenda B. Michira, Qiumin Lu, Juan Li

**Affiliations:** 1 Medical College of Tianjin University, Tianjin University, Tianjin 300072, China; kx_1998@tju.edu.cn; 2 Engineering Laboratory of Peptides of Chinese Academy of Sciences, Key Laboratory of Bioactive Peptides of Yunnan Province, KIZ-CUHK Joint Laboratory of Bioresources and Molecular Research in Common Diseases, National Resource Center for Non-Human Primates, National Research Facility for Phenotypic & Genetic Analysis of Model Animals (Primate Facility), Key Laboratory of Genetic Evolution & Animal Models, Sino-African Joint Research Center, and New Cornerstone Science Laboratory, Kunming Institute of Zoology, The Chinese Academy of Sciences, No.17 Longxin Road, Kunming 650201, China; jams@mail.kiz.ac.cn (J.M.); 2021204059@stu.njau.edu.cn (K.C.); gaojinai18@mails.ucas.ac.cn (J.G.); yangmin@mail.kiz.ac.cn (M.Y.); brendamichira@gmail.com (B.B.M.); 3 Kunming College of Life Science, University of Chinese Academy of Sciences, Kunming 650204, China; 4 College of Life Sciences, Nanjing Agricultural University, Nanjing 210095, China; 5 Center for Evolution and Conservation Biology, Southern Marine Science and Engineering Guangdong Laboratory (Guangzhou), Guangzhou 511458, China; 13477288321@163.com; 6 School of Molecular Medicine, Hangzhou Institute for Advanced Study, University of Chinese Academy of Sciences, Hangzhou 310024, China

**Keywords:** lynx spider, transcriptome, venom-derived peptide, molecular diversification, antimicrobial activity, *Staphylococcus aureus*

## Abstract

Spider venoms are emerging as a rich source of bioactive peptide toxins with therapeutic potential. Lynx spiders of the genus *Oxyopes* are small, cursorial hunters that employ complex venom to subdue arthropod prey. However, extracting crude venom from these diminutive arachnids poses significant challenges. This study presents a transcriptome analysis of venom glands from an undescribed *Oxyopes forcipiformis* species, revealing 339 putative protein and peptide toxin sequences categorized into seven functional groups. The venom composition was dominated by membrane-active peptides (40.71%), venom auxiliary proteins (22.71%), neurotoxins (15.63%), channel active peptides (7.08%) and uncharacterized components (13.87%). Additionally, phylogenetic analysis of 65 disulfide-bond-rich peptides yielded six distinct families based on sequence homology and cysteine framework. Finally, a novel antimicrobial peptide, GK37, was identified using in silico and homology analyses. Our data suggested that GK37 presented significant antibacterial activity against Gram-positive bacteria *Staphylococcus aureus* with a minimum inhibitory concentration (MIC) of 1.552 µM by disrupting bacterial membranes. At 4× MICs, GK37 almost showed no hemolytic activity on blood cells or toxicity against Hek293T cells. Our findings provided a basis for targeted studies of the diversity and pharmacological effects of lynx spider peptide. We elucidated a valuable high-throughput approach for obtaining proteins and peptides from small-group spiders.

## 1. Introduction

Spiders are one of the most efficient terrestrial predators due to their intricate and potent venom systems [1]. This remarkable evolutionary adaptation enables them to dominate as arthropod predators and even prey on larger animals such as birds, bats [2], other mammals, fish [3], amphibians (frogs), squamates and reptiles (lizards and snakes) [4]. Studies have revealed that spider venom is a complex mixture of toxins primarily composed of polypeptides alongside low molecular mass organic components, proteins and other components [5]. Spider venoms present an impressive array of anticancer [6], antimicrobial [7] and analgesic activities [8].

Given the 52,188 species of spiders worldwide in http://wsc.nmbe.ch (accessed on 1 August 2024) [9] that have been described so far, the peptide toxins of the majority of spider venoms remain largely uncharacterized despite the discovery of certain spider venom peptides. Previous studies have mainly focused on large groups because it is extremely difficult to obtain large amounts of venom from small-sized groups, thus explaining why the venom peptides of small spiders have been overlooked. The primary research and discovery of novel peptide toxins from spider venoms, particularly within the suborder Mygalomorphae, have largely been made possible through the application of high-performance liquid chromatography (HPLC) separation techniques. Additionally, the Mygalomorphae suborders, including Theraphosidae, Hexathelidae and Cyrtaucheniidae families, are usually easy to obtain considerable quantities of venom from [10]. The venoms of the above groups have been previously described as being rich in natural peptide toxins that act on different types of K^+^, Na^+^, Cl^−^ and Ca^2+^ ion channels. For example, HaTx1, 2, VSTx1 and GSTxSIA were isolated from the venom of the *Grammostola spatula* (Theraphosidae) [11]. The huwentoxin family, including huwentoxin-I [12], huwentoxin-II [13], huwentoxin-V [14] and huwentoxin-IV [15], represents the neurotoxin purified from the venom of the Chinese bird spider *Ornithoctonus huwena* (Theraphosidae).

Analysis of spider toxin diversification using combined transcriptomic, peptidomic and proteomic approaches has been made feasible with advances in technology, facilitating the discovery of venom peptides from small spiders and further aiding the discovery of functional candidates for biotechnological application, pharmacological industry, agribusiness and others [16]. There are more than 20 families of spiders whose toxin diversity has been studied using next-generation sequencing by analyzing the venom gland transcriptome [17]. It is worth noting that ArachnoServer v3.0 (www.arachnoserver.org accessed on 15 March 2018), the first publicly available bioinformatics pipeline for automated identification of toxins in spider venom gland transcriptomes, catalogs 1404 toxins from 97 species [18]. Most importantly, an increasing number of toxins are being identified and reported [19]. However, most studies have paid more attention to the neurotoxic inhibitor cystine knot (ICK), which is a suitable candidate for drugs and bioinsecticides [20], while the occurrence of antimicrobial peptides (AMPs) in spiders is largely unknown.

AMPs derived from venomous animals have attracted considerable attention for the development of novel active pharmaceutical compounds [21]. Spider venom-derived peptides, also known as cytolytic, interact with a variety of biomembranes to form pores using different mechanisms [22]. Undoubtedly, AMPs are a functionally important group of spider venom components that are as potent as neurotoxins [23]. Spiders can be classified as either web hunters or wanderers, depending on whether they construct webs to capture their prey [24]. Interestingly, AMPs have predominantly been identified in the venoms of the wandering retrolateral tibial apophysis clade (RTA clade), with only a few found in other spider groups [25]. The wandering RTA clade mainly includes six families, namely Ctenidae [25], Lycosidae [26], Oxyopidae [27], Pisauridae [28], Zodariidae [29] and Sparassidae [30]. Recently, AMPs have also been described in web-hunter spiders. For example, two novel AMPs (AATX-Ab2a and AATX-Ab3a) have been reported in the venom of the spider *Argiope bruennichi* [31].

Lynx spiders (Oxyopidae) are typical RTA groups and play a crucial role as pest predators in agroforestry ecosystems. In addition to the traditional neurotoxins and cytotoxins, the two-domain modular toxins spiderlings (OtTx1a, 1b, 2a and 2b) [28] and amphipathic peptides with antimicrobial, hemolytic and insecticidal activity (Oxyopinins) [32] have been identified in lynx spider venom. These toxins display both insecticidal and potent antimicrobial activity, and the latter peptide oxyopinins are the typical linear cationic amphipathic peptides from spider venom [33]. However, few transcriptome analyses have revealed the diversity of lynx spider venom peptides.

Here, we report the toxin diversity of lynx spiders by de novo transcriptome assembly for the venom gland of *O. forcipiformis*, whose small size makes venom collection challenging and crude extract fractionation a hard task [34]. The novel toxin peptide GK37 with antibacterial activities was selected based on in silico homology analysis and functional prediction results. Finally, we evaluated the functional assays in vitro antimicrobial efficacy of GK37, including primary sequence prediction, structure determination, investigation of the potential mechanism for the antimicrobial activity, and evaluation of the therapeutic potential of GK37 as a potential treatment for *S. aureus* infection.

## 2. Results

### 2.1. Venom Gland RNA Extracted and Transcriptome Sequenced

Venom glands obtained from twelve adult female *O. forcipiformis* specimens were partitioned into four groups and stored in liquid nitrogen for subsequent RNA extraction. The RNA libraries were subjected to sequencing using an Illumina NovaSeq 6000 platform. Following the removal of low-quality reads, the sequencing process yielded a cumulative total of 223.10 M raw reads (refer to Table S1).

### 2.2. De Novo Transcriptome Assembled and Functional Annotated

**Figure 1:**
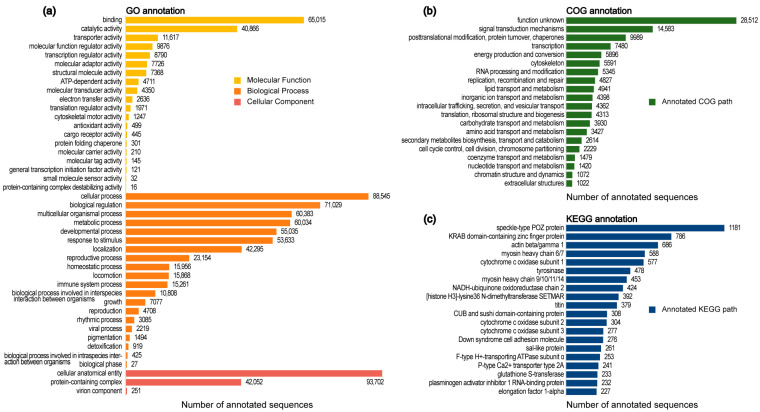
Functional annotation of *O. forcipiformis* assembly. (**a**) GO enrichment of the annotated clean contigs. (**b**) Top 20 functions annotated in COG database of the annotated clean contigs. (**c**) Top 20 pathways of significantly enriched KEGG pathways of the annotated clean contigs.

Transcriptomes for *O. forcipiformis* were constructed based on RNA sequencing data, and the de novo assemblies were generated using Trinity software-v0.23.1 (https://github.com/trinityrnaseq/trinityrnaseq/wiki accessed on 2018). To eliminate redundant transcripts, TGICL (TIGR gene indices clustering tools) was employed, resulting in 218.28 M clean reads. Details of the de novo assembly process are provided in Table S1. A total of 493,538 contigs (ORF) with an average length of 999.83 bp were identified using Trans Decoder software v5.7.0. (https://github.com/TransDecoder/TransDecoder accessed on 16 July 2023). Subsequently, a homology search against various public protein databases was conducted after removing Symbiodinium contamination from the clean contigs (ORF). Specifically, 92,487 (28.98%), 99,318 (31.12%) and 127,368 (39.91%) of the clean contigs showed matches in the Kyoto Encyclopedia of Genes and Genomes (KEGG) (https://www.genome.jp/kegg/ accessed on 1 January 2001), Gene Ontology (GO) (https://www.geneontology.org/ accessed on 8 September 2024) and Clusters of Orthologous Genes (COGs) (https://www.ncbi.nlm.nih.gov/research/cog/ accessed on 2021) databases, respectively, as presented in Table S1 and Figure 1. Furthermore, 5710 transcripts exhibited similarities to venom proteins in the Tox-Prot database of UniProtKB/Swiss-Prot (https://www.uniprot.org/help/Toxins accessed on 18 April 2023) among the identified transcripts.

### 2.3. Putative Venom-Related Polypeptides Identified from the Transcriptome of O. forcipiformis

**Figure 2:**
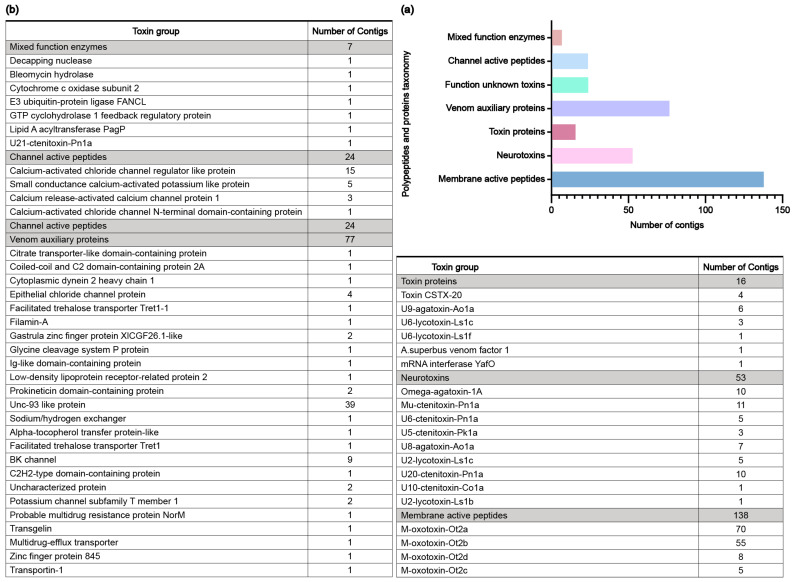
The potential toxin contigs of *O. forcipiformis* identified from the transcriptome. (**a**) Classification of putative polypeptides and proteins identified from transcriptome mining from *O. forcipiformis*. (**b**) Typical toxin families and number of these families in each classification. There are 339 putative protein and peptide toxin sequences that can be categorized into seven functional groups.

Based on annotated results, we predicted putative venom-related polypeptides from *O. forcipiformis*. An additional HMMER search was performed against the Pfam database (http://pfam-legacy.xfam.org/ accessed on 11 October 2023) to further validate the putative polypeptide sequences, resulting in the identification of 339 putative polypeptides. A summary of the number and classification of putative venom-related polypeptides is presented in Figure 2a, while brief descriptions of representative polypeptide sequences are provided in Figure 2b. The potential toxin contigs of *O. forcipiformis* typically encompass various forms of membrane-active peptides, venom auxiliary proteins, neurotoxins and channel-active peptides. Additionally, the analysis and discussion of the potential multiple disulfide bond toxins, which are significant peptides of *O. forcipiformis*, are presented in the subsequent sections.

### 2.4. Phylogenetic Analysis of the Identified Knottins

**Figure 3:**
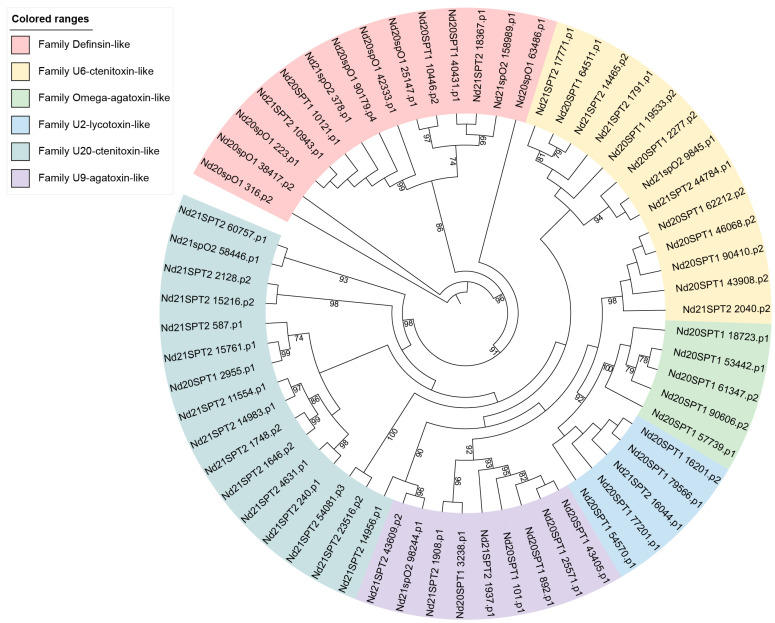
Phylogenetic tree showing putative toxin polypeptides from the venom glands of *O. forcipiformis*. The MEGA 7.0 software (https://www.megasoftware.net/ accessed on 22 March 2016) package was used to conduct phylogenetic analysis using the neighbor-joining method. The tree features six superfamilies, each distinguished by color coding. Phylogenetic tree analysis revealed the classification of *O. forcipiformis* toxin polypeptides into 6 superfamilies.

Utilizing the BLAST+ package (v2.13.0) (https://www.ncbi.nlm.nih.gov/ accessed on 29 March 2022) offered by NCBI, a screening of 65 sequences rich in disulfide bonds derived from toxins was conducted using the UniProt database. These sequences were categorized into families according to their sequence homology. The phylogenetic tree analysis led to the classification of *O. forcipiformis* toxin polypeptides into six superfamilies (refer to Figure 3).

Sequence comparisons with the NR protein database indicated that the potential toxin polypeptides within superfamilies exhibited significant similarity to toxin peptides derived from the African social velvet spider *Stegodyphus mimosarum* [35]. Defensin displayed a notable sequence resemblance to *O. forcipiformis* peptide toxins. Additionally, toxins from the orb-weaver spider *Araneus ventricosus* [36] also showed a high sequence similarity to *O. forcipiformis* toxins. Alignment of the sequences with the UniProt database revealed that the putative toxin polypeptides shared a high degree of similarity with neurotoxins that act as activators/blockers of Na, K and Ca ion channels.

### 2.5. Family Analysis of Knottins in O. forcipiformis

**Figure 4:**
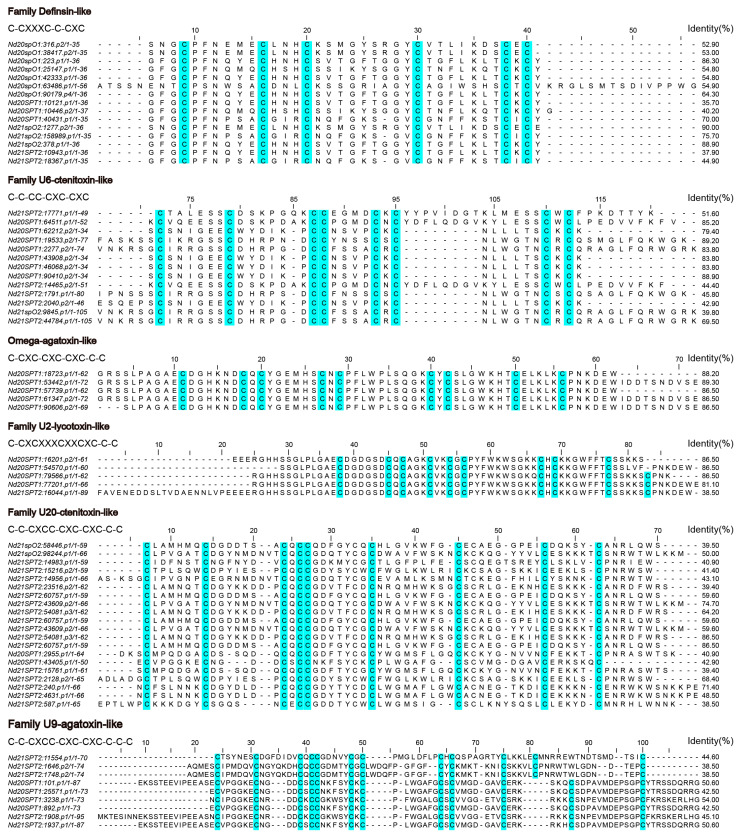
Multiple alignment analysis of the transcriptomic RNA-deduced amino acid sequences for putative toxins of *O. forcipiformis*. These sequences have been grouped into 6 families according to their sequence homology. Conserved cysteines are highlighted in blue.

We classified 65 disulfide-bond-rich toxin polypeptide sequences into six groups based on the mature peptides’ number and alignment pattern of cysteines in Figure 4. We found that knottins, mainly from six different gene superfamilies in varying proportions, were remarkably specific.

#### 2.5.1. Family Definsin-like

Family Definsin-like had 15 toxin polypeptides, and all predicted mature peptides containing the same cysteine framework, C1-C2-C3-C4-C5-C6. The Family Definsin-like toxin group had moderate homology (45.8–70.0% similarity) with the peptide toxin Definsin [35] from the African social velvet spider *Stegodyphus mimosarum* based on the NR and UniProt protein databases. Its predicted biological process is a defense response to bacterium and has an antimicrobial function.

#### 2.5.2. Family U6-Ctenitoxin-like

Thirteen toxin polypeptides belonging to Family U6-ctenitoxin-like shared the same cysteine framework and very comparable mature peptide amino acid sequences, C1-C2-C3C4-C5-C6-C7-C8. The toxin precursor peptides were divided into three groups: (1) Five sequences (39.4% similarity) best matched to the toxin U6-ctenitoxin-Pn1a [37] sequence from the Brazilian armed spider *Phoneutria nigriventer*, which showed function as antagonist of L-type calcium channels (Cav1/CACNA1); (2) a sequence homology (31.2–37.3% similarity) with the U6-lycotoxin-Ls1c [38] sequence from the wolf spider *Lycosa singoriensis* of three sequences had toxin function; (3) the other toxins best matched to U8-agatoxin-Ao1a [36] from the orb weaver spider *Araneus ventricosus* (59.6–71.4% similarity) were predicted to have ion channel inhibitor activity.

#### 2.5.3. Family Omega-Agatoxin-like

Family Omega-agatoxin-like consists of five toxin polypeptides, all of which share a common cysteine framework: C1-C2-C3-C4-C5-C6-C7-C8-C9. These toxins show moderate similarity (39.8–50.0%) to the peptide toxin Omega-agatoxin-1A [39] from the North American funnel-web spider *Agelenopsis aperta* according to the NR and UniProt protein databases. The toxins in this family are antagonists of voltage-gated calcium channels and block insect neuromuscular transmission presynaptically. Specifically, they act as blockers of L-type calcium channels (Cav/CACNA1). This suggests that Family Omega-agatoxin-like toxins may play a significant role in inhibiting channels and aiding in insect predation.

#### 2.5.4. Family U2-Lycotoxin-like

Family U2-lycotoxin-like had five toxin polypeptides, with a consistent cysteine framework, C1-C2-C3-C4-C5-C6-C7-C8-C9-C10. These polypeptides can be divided into two groups: (1) Four sequences with a 45.2–51.6% sequence similarity with U2-lycotoxin-Ls1c [40] from the wolf spider *Lycosa singoriensis*, which has insecticidal to house crickets and causes slow-onset excitatory effects leading to irreversible spastic paralysis. This toxin also modulates the human voltage-gated potassium channel Kv1.5/KCNA5, likely binding to the voltage-sensing domain of the channel, preventing its opening at physiological membrane potentials. The recombinant peptide binds irreversibly to the channel, slowing down the activation kinetics of the hKv1.5 current. It is not toxic to mice when injected intracranially at a dose of 0.5 μg/g mouse (20 ug). (2) We predicted the maturation peptide last with a 48.4% sequence similarity with Omega-agatoxin-1A [39] from the North American funnel-web spider *Agelenopsis aperta*. Omega-agatoxin-1A activated the calcium channel voltage, suggesting that toxins in *O. forcipiformis* may also have Cav channel activity.

#### 2.5.5. Family U20-Ctenitoxin-like

Family U20-ctenitoxin-like was the most abundant superfamily, with a consistent cysteine framework (C1-C2-C3-C4C5-C6-C7-C8-C9-C10-C11) and contained 18 non-redundant toxin precursor peptides that were categorized into four groups: (1) Six sequences exhibited sequence similarity with the toxin Mu-ctenitoxin-Pn1a [41] from the Brazilian armed spider *Phoneutria nigriventer* (34.7–47.1% similarity). Mu-ctenitoxin-Pn1a is a reversible inhibitor of neuronal sodium channels (Nav1.2/SCN2A), binding near site 1 and exhibiting increased affinity as the membrane potential becomes depolarized. It induces excitatory symptoms and spastic paralysis in mice, suggesting that Family U20-ctenitoxin-like toxins may play an important role in channel activation. (2) A total of 11 sequences best matched with U20-ctenitoxin-Pn1a [42] (38.5–44.6% identity) from the Brazilian armed spider *Phoneutria nigriventer*. It acts as a biological antagonist of voltage-gated calcium channels (Cav), and injection of 3 ug per mouse (20 ug)into the cerebral ventricle of mice induces general flaccid paralysis and immediate death. (3) In this group, one mature peptide sequence had homology (42.9% similarity) with U5-ctenitoxin-Pk1a [43] from the Brazilian wandering spider *Phoneutria keyserlingi*. U5-ctenitoxin-Pk1a is a deadly neurotoxin that causes spastic paralysis and kills mice within 4–6 min of intracerebroventricular injection at 1.5 ug per mouse (20 ug). (4) In the fourth group, the toxin peptide had homology (54.7% similarity) with U9-agatoxin-Ao1a [44] from the Funnel-web spider *Agelena orientalis*, which showed toxin activity.

#### 2.5.6. Family U9-Agatoxin-like

Family U9-agatoxin-like had eight toxin polypeptides, with a consistent cysteine framework, C1-C2-C3-C4C5-C6-C7-C8-C9-C10-C11-C12. The eight toxin precursor peptides were divided into two groups: (1) Two sequences exhibited sequence similarity (38.5%) with the toxin Mu-ctenitoxin-Pn1a as Family U20-ctenitoxin-like group (1). (2) The other six toxins best matched to U9-agatoxin-Ao1a as family U20-ctenitoxin-like group (4) with 42.5–54.0% similarity.

### 2.6. GK37 Identified as a Novel Antimicrobial Peptide by Analysis of Putative Toxin Polypeptides

The AI-based result of the prediction showed that 16 have peptides antimicrobial properties (Table S5). Next, we found that a segment of a transcript (ID: Nd20SPT1: 79745.p1) showed 67.6% similarity to a venom-derived neuropeptide M-oxotoxin-Ot2b [45] (UniProt ID: P83248) obtained from the lynx spider *Oxyopes takobius* via a NCBI-BLASTP (https://blast.ncbi.nlm.nih.gov/Blast.cgi accessed on 9 October 2023) search.

**Figure 5:**
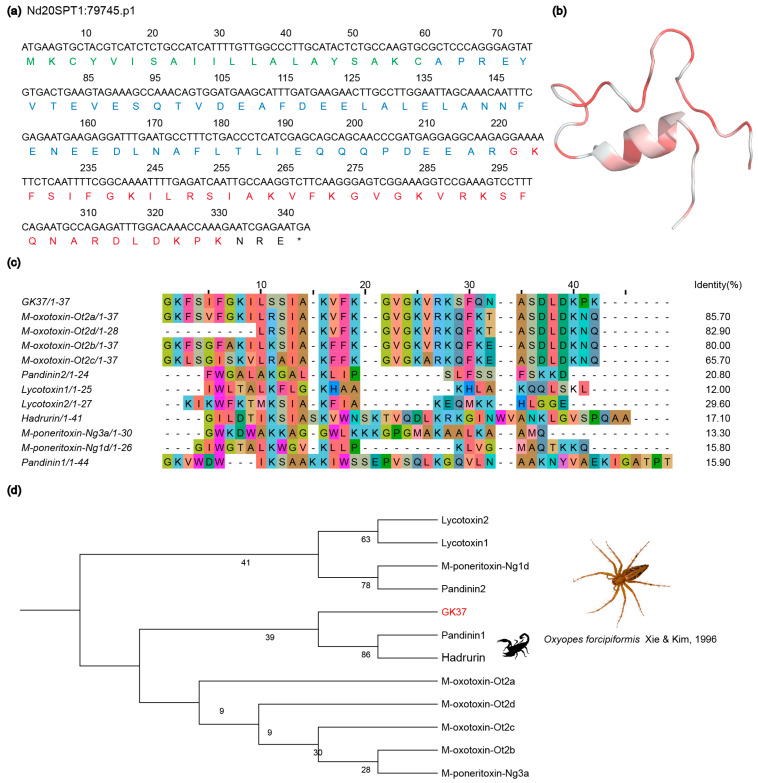
Multiple sequence alignment and structure analysis of GK37. (**a**) DNA nucleotide and deduced amino acid sequences of GK37 (Nd20SPT1:79745. p1) are shown in full length. The putative signal peptides are highlighted in green, the pro-peptide is represented in blue, and the mature peptide GK37 is shown in red. (**b**) The predicted tridimensional structure of GK37 was based on the peptide sequence. Red: hydrophobic amino acids; white: hydrophilicity amino acids. (**c**) Multiple amino acid sequence alignment of GK37 peptides and similar peptides from various species. (**d**) A maximum-likelihood tree is generated from phylogenetic analysis, comparing GK37 (highlighted in red) with different similar peptides.

The M-oxotoxin-Ot2b demonstrated antimicrobial properties against the Gram-negative bacterium *Escherichia coli* (*E. coli*), Gram-positive bacteria *Bacillus subtilis* (*B. subtilis*) and *S. aureus*. It also exhibited insecticidal effects on *Spodoptera frugiperda* ovarian cells by activating non-selective ion channels and enhancing the insecticidal activity of spider venom neurotoxic peptides. Consequently, the entire mature domain GKFSIFGKILSSIAKVFKGVGKVRKSFQNASDLDKP of this transcript was isolated and labeled as GK37 for subsequent analysis, as illustrated in Figure 5a, which includes multiple sequence alignment and phylogenetic examination. Noteworthy is the fact that GK37 consists of 37 amino acids with a molecular mass of 4026.73, as shown in Figure S1. The Alphafold 2 website was utilized to predict the tridimensional helical structure of GK37, depicted in Figure 5b. Circular dichroism (CD) analysis confirmed the amphiphilic nature of GK37. The peptide displayed an increased helical content in trifluoroethanol (TFE) compared with phosphate-buffered saline (PBS), indicating structural adaptability. This alteration in conformation and amphiphilic properties is likely pivotal in the bactericidal function of GK37.

Further multiple sequence alignment and phylogenetic analysis revealed that GK37 exhibited similarities with various arthropod antimicrobial peptide analogs, such as pandinins from *Pandinus imperator* [46], lycotoxins from *L. carolinensis* [47], hadrurin from *Hadrurus aztecus* [48] and M-poneritoxin from *Neoponera commutata* [49]. Notably, GK37 clustered with the pandinins toxins, which are α-linear antimicrobial peptides found in the venom of the scorpion *P. imperator*. Pandinin 1 was characterized as a low hemolytic and antimicrobial peptide, while Pandinin 2 was identified as a highly hemolytic and antimicrobial peptide, sharing biological and structural similarities with melittin. Therefore, we also predicted the hemolytic activity of GK37 by HLPpredfuse (http://thegleelab.org/HLPpred-Fuse/index.html accessed on 14 April 2020) and HemoPi 2.0 (https://webs.iiitd.edu.in/raghava/hemopi/batch.php accessed on 2 July 2020) (Figure S5). The results showed Gk37 has a 71.979% probability of high hemolytic activity, and the PROB score was 0.54, which means it has a normalized SVM score and ranges between 0 and 1 (i.e., a score of 1 is very likely to be hemolytic and 0 is very unlikely to be hemolytic).

### 2.7. GK37 Exerted Antimicrobial Activity Against S. aureus

**Figure 6:**
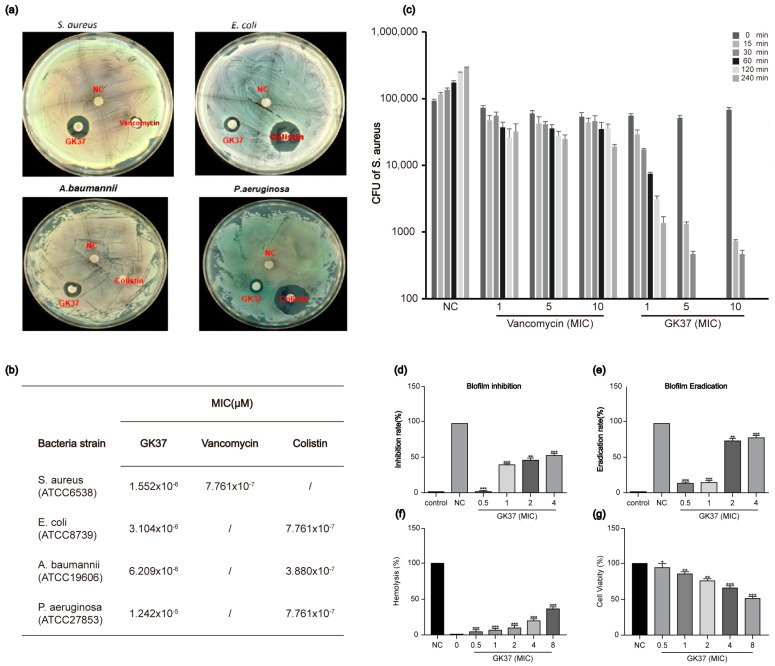
The antibacterial activity and safety of GK37. (**a**) Result of the zones of inhibition formed by GK37 against *S. aureus*, *E. coli*, *A. baumannii* and *P. aeruginosa*. (**b**) Antimicrobial activity of the peptides against four standard bacterial strains. (**c**) Killing kinetics assay of GK37 against *S. aureus*. (**d**) Inhibitory effects of GK37 on the biofilm formation of *S. aureus*. (**e**) The effects of GK37 on the established biofilm eradication of *S. aureus*. (**f**) The hemolytic activity of GK37 on human erythrocytes. (**g**) The survival rate of HEK293T with the peptide GK37. NC: saline, GK37: 10 mg/mL, vancomycin: 10 mg/mL, Colistin: 10 mg/mL. Data represent means ± SD of 3 independent experiments. * *p* < 0.05, ** *p* < 0.01, *** *p* < 0.001.

To assess GK37’s antimicrobial potential, a chemically synthesized peptide was used, and its inhibition zones and MICs against four standard strains were confirmed. As shown in Figure 6a, GK37 formed zones of inhibition against *S. aureus* ATCC6538, *Escherichia coli* ATCC8739, *Acinetobacter baumannii* ATCC19606 and *Pseudomonas aeruginosa* ATCC27853, and showed potent antibacterial activity in Figure 6b. We chose *S. aureus* ATCC6538 as a study model in the subsequent mechanism research.

#### 2.7.1. GK37 Exhibited Rapid Killing Ability Against *S. aureus*

The bactericidal efficacy of GK37 against *S. aureus* was tested alongside vancomycin, one classic antibiotic for treating *S. aureus* [38], by a time-kinetic killing assay in Figure 6c. Compared to vancomycin, GK37 exhibited a time-dependent and dose-dependent antibacterial performance. At 1× MIC, GK37 demonstrated bactericidal ability with 100% lethality over time up to 240 min, while vancomycin only showed limited ability with a 74.5% survival rate. Similarly, the peptide killed all bacteria within 60 and 30 min at 5 and 10× MICs, respectively. The efficient bactericidal kinetic results of GK37 indicated that although GK37 was less active than vancomycin, it might kill bacteria by rapid membrane perturbation.

#### 2.7.2. GK37 Exhibited Biofilm Inhibition and Eradication Activities

For antibacterial biofilm experiments, we checked the effectiveness of GK37 in inhibiting and eradicating biofilm formation of *S. aureus* at various concentrations (0.5, 1, 2 and 4 × MICs). The results show that GK37 significantly inhibited *S. aureus* biofilm in a concentration-dependent manner. At 2× MICs, GK37 reduced biofilm formation by approximately 50% in Figure 6d and removed 74% of the established one in Figure 6e. This great efficacy indicated that the antimicrobial mechanism of GK37 might involve targeting both biofilm and planktonic cells [50].

#### 2.7.3. GK37 Showed Almost No Hemolytic Activity or Cytotoxicity

Based on the prediction, the results show that the GK37 had hemolytic activity probability, and we performed hemolysis assays to confirm the safety of GK37 for drug use. As illustrated in Figure 6f, GK37 performed low hemolytic activity of 23% hemolysis rate at 4× MICs compared with the negative control. Additionally, we evaluated the cytotoxicity of GK37 against HEK293T cells. Results show that the survival rate of HEK293T cells is approximately 70%, even at 4× MICs in Figure 6g. These data suggest that GK37 is within the safe and effective range for antimicrobial use.

#### 2.7.4. GK37 Maintained Antibacterial Activity in Plasma

Co-incubation assay was used to confirm the stability of GK37 in human plasma. The result suggests that GK37 in human plasma still maintained its structure and antimicrobial activity against *S. aureus* used at 1.552 μM (Table S4).

### 2.8. GK37 Exhibited Antimicrobial Activity by Cell Membrane Permeabilization

**Figure 7:**
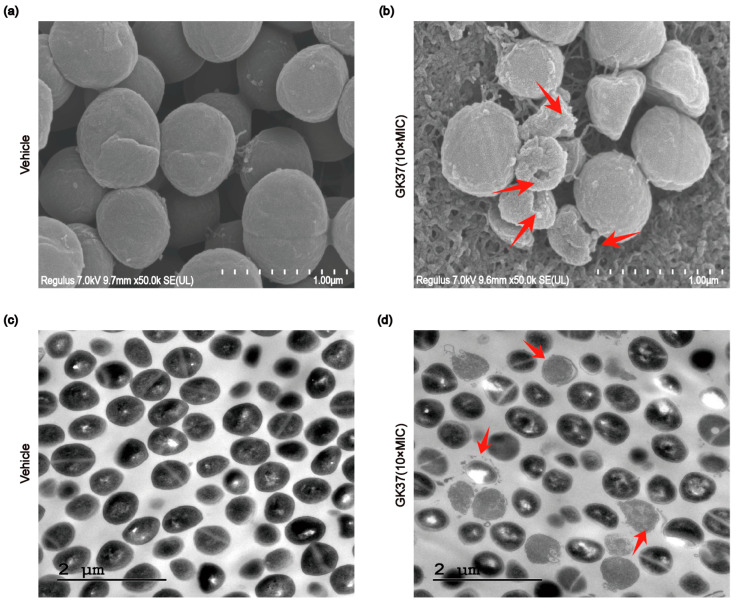
The membrane morphology changes of *S. aureus* treated with GK37 (10× MICs) for 2 h determined by SEM and TEM. (**a**) The morphology of untreated *S. aureus* under SEM. (**b**) The morphology of *S. aureus* interacting with GK37 under SEM. Red arrows point to the distorted and shriveled bacterial membrane. (**c**) The morphology of untreated *S. aureus* under TEM. (**d**) The morphology of *S. aureus* interacting with GK37 under TEM. Red arrows point to the membrane lysis area.

Next, we employed transmission electron microscopy (TEM) and scanning electron microscopy (SEM) to examine the morphology changes of *S. aureus* induced by 10× MICs of GK37. Images clearly show that the surface of untreated *S. aureus* appeared smooth and had a round or oval outline. However, after interacting with GK37, significant changes were observed within distorted and shriveled bacterial membranes and cytoplasmic lysis in Figure 7a–d. Thus, those findings provide evidence that GK37 exhibited potent antibacterial activity against *S. aureus* by membrane perturbation mechanism.

## 3. Discussion

Numerous highly bioactive peptides have been isolated from spider venom, demonstrating anticancer, antimicrobial, and analgesic activities [4]. Functional assays of bioactivity are primarily facilitated by HPLC separation, which aids in isolating bioactive peptides from spider venoms. However, given the small size of spiders and little venom secretion, getting sufficient venom amounts for detailed analysis is extremely challenging. For this reason, previous studies have always been limited to large groups such as Theraphosidae, Hexathelidae and Cyrtaucheniidae [10]. Nonetheless, spider venoms are increasingly recognized as a treasure trove for drug therapy. Furthermore, leveraging advanced bioinformatics, the unexplored diversity of spider venoms is expected to uncover novel peptides with distinct functions. To date, more than 20 families of spiders have been analyzed for peptide diversity using high-throughput screening techniques, transcriptomics and proteomics [17]. Recent technological and methodological advances, such as breakthroughs in synthetic biology and peptide synthesis, have played a pivotal role in exploring the novel functionalities of novel peptides. The growing accessibility of high-throughput transcriptomic data creates opportunities to discover and characterize novel peptides with valuable bioactivities from previously unexplored spider venom glands.

In this study, we conducted a comprehensive analysis of the predicted toxin repertoire of the lynx spider *O. forcipiformis*, revealing the diversity of lynx spider venom peptides. AMPs have gained attention as a new source of antimicrobial due to their efficacy against multi-drug-resistant pathogens with fewer side effects [51]. In addition, the novel AMPs GK37 with significant antimicrobial activity against both Gram-negative and Gram-positive bacteria such as *S. aureus*, *E. coli*, *A. baumannii* and *P. aeruginosa* were identified from venom gland transcripts of the spider *O. forcipiformis* using bioinformatics. The transcriptome data have been deposited at CNCB (China National Center for Bioinformation). To the best of our knowledge, this study is the first to utilize de novo transcriptomic to identify the peptide toxin repertoire of *O. forcipiformis*, contributing to our understanding of venom diversity in smaller spider species.

Initially, we conducted de novo transcriptome assembly and annotation to characterize the venom composition of *O. forcipiformis*. Our customized bioinformatic pipeline identified 339 toxin polypeptides across seven categories. The three most abundant families were membrane-active peptides (40.71%), venom auxiliary proteins (22.71%) and neurotoxins (15.63%). In addition, phylogenetic analysis of the 65 identified knottins revealed that these polypeptides in superfamilies A, B, C, D, E and F were highly similar to the toxin peptides of the African social velvet spider *S. mimosarum* and the orb weaver spider *A. ventricosus*. In summary, we revealed that the putative toxin polypeptides of *O. forcipiformis* had a high similarity to the neurotoxins with Na, K, and Ca ion channel activators/blockers. Some novel peptide sequences have evolved independently, highlighting the sequence diversity of the *O. forcipiformis* toxins. This study increased the number of superfamilies in the spider toxin library and offered predictive value for a better understanding of the relationship between species consanguinity and spider toxin evolution.

Secondly, our investigation of potential toxin polypeptides resulted in the discovery of a new antimicrobial peptide, GK37. While previous research on spider venom polypeptides has predominantly concentrated on neurotoxins and cardiotoxins that target various ion channels [52], our focus was to examine overlooked polypeptides with unique functions, such as antimicrobial peptides in *O. forcipiformis*. Given the escalating threat of antibiotic resistance, there is an urgent need for novel antimicrobial agents. Spider venom, a complex chemical blend abundant in biologically active peptides [53], harbors numerous natural antimicrobial peptides that could potentially be harnessed as alternatives to antibiotics [54]. For example, Lycosin-I, derived from the venom of *L. singoriensis*, exhibits a broad-spectrum antibacterial property with effective antibacterial action at micromolar concentrations, and it demonstrates synergistic antibacterial effects when combined with conventional chemical antibiotics [55]. Additionally, Lycosin-I exerts noticeable inhibitory effects on the growth of cancer cell lines and tumors in vivo by impeding cell proliferation or triggering apoptosis [56]. In essence, Lycosin-I represents a new antimicrobial and antitumor peptide with promising prospects for pharmaceutical development, underscoring the likelihood that spider antimicrobial peptides will emerge as a crucial reservoir of innovative antimicrobial agents [57]. Consequently, our study identified a novel antimicrobial peptide (GK37) from *O. forcipiformis* for subsequent in silico and virtual screening. Specifically, GK37 consists of 37 amino acid residues and exhibits a helical structure with hydrophilic/hydrophobic terminal regions. Comparative sequence alignment and phylogenetic analysis indicated that GK37 is an arthropod antimicrobial peptide analog, sharing resemblances with the pandinin toxin (a low hemolytic and antimicrobial peptide) from *P. imperator* and the hadrurin toxin from *H. aztecus*.

The prediction of biological activity based on the primary sequence has limitations, necessitating the verification of precise biological activity through in vitro testing using the toxin monomer. Our data indicated that GK37 demonstrated significant antimicrobial activity against both Gram-negative and Gram-positive bacteria, such as *S. aureus*, *E. coli*, *A. baumannii* and *P. aeruginosa*. Using *S. aureus* as a model organism, the biological activity and antimicrobial mechanisms of GK37 were further validated, revealing its rapid and direct bactericidal effects against *S. aureus* mechanism of GK37 through perturbation of the bacterial membrane. However, these data only suggested the membranolytic effect as one possibility, and we cannot exclude other possible active mechanisms. Furthermore, the peptide exhibited potent antibiofilm activity, inhibiting the formation and eradicating preformed *S. aureus* biofilms. Additionally, GK37 exhibited good plasma stability, low cytotoxicity and hemolytic activity within the effective range of 4 × MICs. In conclusion, these results underscored the excellent antibacterial properties of GK37, indicating its potential for further academic investigation and therapeutic applications. In further work, we will study if bacteria develop resistance against this novel peptide or not and test it at higher concentrations in hemolysis and cytotoxicity assays to make sure of its safety to humans.

On the other hand, we are also interested in the prediction results of both AI tools that classified GK37 as a potential hemolytic peptide, which did not match the experimental data. We think this result may be based on the large amount of data on venom hemolytic peptides, like Pandinin 1 and Pandinin 2. In the era of AI-based technologies, it is important to evaluate in silico tools, which are data-dependent. Other studies also suggested that more optimization work has to be done in order to attain bioinformatics approaches with high accuracy and minimize mistakes [58]. Therefore, this study can serve as a reminder that using the models to accelerate drug discovery should continuously evaluate the silico tools.

## 4. Conclusions

In conclusion, we have established the foundation for the identification of multiple possible biological tools from the venom of *O. forcipiformis*, as well as added a number of new members to the spider toxin superfamily. Additionally, the characterization of the *O. forcipiformis* venom toxins provided new insights into venom composition, revealed the potential mechanism of action of venom peptides and identified GK37 as a novel and potent AMP against *S. aureus*. These findings underscore the significance of *O. forcipiformis* venom toxins as prospective sources for the development of pharmacological and antimicrobial drugs and suggest that GK37 is a prime candidate or template for creating therapeutic agents to treat *S. aureus* infections.

## 5. Materials and Methods

### 5.1. Sample Procession of O. forcipiformis

The *O. forcipiformis* specimen was collected from Daxi Mountain, Nansha City, Guangzhou Province, China. Venom gland samples were obtained from 12 female spiders and cryopreserved in liquid nitrogen. Total RNA was extracted from the venom gland by Trizol following the manufacturer’s instructions (Invitrogen Life Technologies, Carlsbad, CA, USA), and the quality of the RNA was analyzed by agarose gel electrophoresis and assessed on an Agilent 5400 system (Agilent, CA, USA) [59].

### 5.2. Construction of RNA Library and Transcriptome Sequencing

The prepared samples were sent to Beijing Kinko Biotechnology Co., Ltd. for transcriptome sequencing (based on the Illumina Hiseq platform, using the Illumina Truseq TM RNA sample prep kit method to construct four libraries) [60]. High-quality clean reads were obtained by quality control (FastQC) and filter (Trimmomatic) [61].

### 5.3. Data Procession and Functional Annotation

Using Trinity software-v0.23.1 with default parameters [62], the clean data were de novo spliced and assembled to obtain the transcripts, and the longest sequences of each gene cluster were filtered out to be used as the UniGene for the subsequent analysis [63]. We annotated the UniGene and transcript obtained after assembly in NCBI, KEGG, GO, KOG and Pfam databases, as well as Diamond BLASTp to search Nr and Swiss-Prot databases for comprehensive functional gene annotation based on sequence similarity [64].

### 5.4. Polypeptides Identification from O. forcipiformis

The transcripts were analyzed using BLASTp to search an exclusive database for toxins and venom proteins in the animal toxin annotation project (Tox-Prot) in order to find any probable venom-related polypeptides. Furthermore, the online program Signalp 6.0 [65] was utilized to confirm the presence of a signal peptide in each of the inferred venom-related polypeptides. Spider toxin pre-peptides were predicted by SpiderP ArachnoServer [66]. Adobe Photoshop 2020 and Adobe Illustrator 2020 were used for graphical processing.

### 5.5. Multiple Sequence Alignment and Phylogenetic Detection of O. forcipiformis Neuropeptide

Multiple sequence alignment was carried out using the Clustal Omega program server, and the results were displayed using Jalview software 2.11.2.0 [67]. Using the neighbor-join approach, phylogenetic analysis was carried out using MEGA software X and the MUSCLE algorithm [68]. Finally, the bootstrap method with 700 replicates was used to assess the phylogenetic tree’s reliability.

### 5.6. In Silico Analysis of Haemolysis and Antimicrobial Properties

We predicted the antimicrobial peptides from the venom glands transcriptome of the spider *O. forcipiformis* using TIDETRON COMDEL (https://ai.tidetronbio.com accessed on 9 November 2023). Additionally, we predicted the probability of hemolytic activity of GK37 by two web servers: HLPpredfuse and HemoPi.

### 5.7. Preparation of Peptide

The GK37 peptide was synthesized using solid-phase chemistry by Hangzhou Gutuo Biotechnology Co. Ltd. Analytical reverse-phase HPLC (RP-HPLC) and mass spectrometry (MS) confirmed the peptide’s purity, which was 96.20% and was characterized by a single peak. Complete deprotection and cleavage were carried out using trifluoroacetic acid (TFA) in water. The mobile phase was composed of 0.1% TFA in water (A) and acetonitrile (B). The ESI-MS was operated in positive ion mode. The peptides were obtained in solid powder form and dissolved in physiological saline to prepare stock solutions.

### 5.8. Bacterial Strain Preparation and Growth Conditions

The bacterial strains preserved in glycerol and used in this study included *P. aeruginosa* (ATCC27853), *A. baumannii* (ATCC19606), *E. coli* (ATCC8739) and *S. aureus* (ATCC6538). All strains were identified and verified by Kunming Prime Biology and cultured in Luria-Bertani (LB) broth.

### 5.9. Detection of In Vitro Antimicrobial Ability

For the inhibition zone diameter experiment, 10 μL of 2 × 10^8^ CFU/mL bacterial suspension was spread evenly on a solid medium. Filter paper discs were then treated with 10 μL of GK37 and positive controls at a concentration of 10 mg/mL, with PBS serving as the negative control. The samples were incubated overnight at 37 °C, and the diameters of the inhibition zones were measured.

To determine the minimum inhibitory concentration (MIC), the bacterial inoculum was diluted to 2 × 10^5^ CFU/mL, and 100 μL of the dilution was transferred into a 96-well plate. The bacterial suspension was then mixed with an equal volume of increasing peptide concentrations (0.062 to 15.892 μM) and incubated at 37 °C. After overnight incubation, the absorbance was measured using a microplate reader at 600 nm (Molecular Devices, Sunnyvale, CA, USA). The MIC was identified as the lowest concentration of peptide that inhibited visible bacterial growth [69].

### 5.10. Determination of Bacterial Killing Kinetic

The bacterial killing kinetics assay was conducted based on a previously described method with slight modifications [70]. *S. aureus* was grown to the exponential phase and diluted to 1 × 10^5^ CFU/mL in RPMI 1640 medium with 10% FBS. GK37 (at 1, 5 and 10 times the MIC) or vancomycin (at the same concentrations) was added to the bacterial suspension, which was incubated at 37 °C for various time intervals (0, 15, 30, 60, 120 and 240 min). At each time point, 10 μL aliquots were taken, diluted 1000-fold with saline, and 100 μL of the dilution was plated on agar plates. Following a 24 h incubation at 37 °C, the number of viable colonies was counted.

### 5.11. Inhibition and Eradication of Biofilm Assay

To assess the anti-biofilm activity of GK37, a biofilm inhibition assay was conducted with slight adjustments based on previous research [70]. In brief, *S. aureus* at a concentration of 2 × 10^6^ CFU/mL was cultured in 96-well plates containing 200 μL of RPMI 1640 medium with 10% FBS, incubated at 37 °C for 24 h. The plates were divided into groups with or without GK37, added at concentrations of 0.5, 1, 2 and 4 times the MIC. After 24 h, the plates were washed thrice with sterile PBS to remove any planktonic bacteria. The remaining biofilms were fixed with 99% methanol for 15 min and stained with 0.1% crystal violet for 5 min. The excess stain was gently rinsed with water, and the dye was dissolved in 95% ethanol. The absorbance at 600 nm was measured to quantify the biofilm biomass. For the eradication of preformed biofilms, *S. aureus* biofilms were grown in a 96-well plate by adding 200 μL of bacteria at a concentration of 1 × 10^6^ CFU/mL in RPMI 1640 medium with 10% FBS, followed by incubation at 37 °C for 24 h. The biofilms were washed three times with PBS, and GK37 at various concentrations (0.5, 1, 2 and 4 times the MIC) was added to the wells containing the biofilms, while negative control wells received media without the peptide. After a 24 h incubation at 37 °C, the wells were emptied, and the eradication of the biofilms was quantified as described above.

### 5.12. Evaluation of Bacterial Membrane Morphology

Bacterial membrane morphology was observed using SEM and TEM following previous research with minor modifications [71]. *S. aureus* was cultured in LB broth to the exponential phase, washed twice with saline, and then incubated with or without GK37 at 10 times the MIC for 2 h at 37 °C. The suspension was centrifuged at 3500 rpm for 5 min, and the bacterial pellets were fixed in 2.5% buffered glutaraldehyde at 4 °C for 12 h. The pellets were then embedded in agar, sliced and vacuum-sputter coated with gold. Bacterial morphology changes were observed under SEM and TEM, and images were captured at appropriate magnifications.

### 5.13. Discussion of Hemolysis and Cytotoxicity

To assess the potential side effects of GK37, hemolysis assays were conducted based on prior reports with slight modifications. For the hemolysis assay, 100 μL of human red blood cell suspension was incubated with 100 μL of GK37 at varying concentrations (0 to 24.832 μM) for 30 min at 37 °C. After centrifugation, the absorbance of the supernatant was measured at 450 nm. Saline was used as the “zero hemolysis” control, while 1% (*v*/*v*) Triton X-100 represented 100% hemolysis. The hemolysis induced by the sample was calculated as a percentage relative to Triton X-100-induced hemolysis.

Human HEK293 embryonic cells (1 × 10^5^ cells/mL) from the Kunming Institute of Zoology, Chinese Academy of Sciences Cell Bank were used to assess cytotoxicity. The cells were cultivated at 37 °C in a humidified atmosphere with 5% CO2 in 96-well plates using Dulbecco’s Modified Eagle’s Medium (DMEM, Gibco, GrandIsland, NY, USA), which included 10% FBS and penicillin (100 U/mL)-streptomycin (100 µg/mL). After 24 h, fresh medium with or without the test peptides at various concentrations was added to the wells, and incubation continued for another 24 h. CCK8 was added to each well, followed by further incubation for 2–4 h. Absorbance at 450 nm was measured using a multifunctional microplate reader. Blank control wells received only the F12 culture medium, negative control wells contained the F12 culture medium, and positive control wells contained the F12 culture medium with 10% DMSO.

### 5.14. Variation in Antimicrobial Activity of GK37 in Plasma

We used a previously reported method [72] to determine the stability of GK37 in plasma. Briefly, an equal volume of GK37 was mixed with human plasma and diluted to a concentration of 24.832 μM. After incubation at 37 °C at different time points (0, 0.5, 1, 2, 4, 6, 8 and 12 h), the MICs of GK37 against *S. aureus* were determined.

### 5.15. Statistical Analysis

Quantitative data were presented as the mean ± standard deviation (SD) and plotted with GraphPad Prism version 9.0 (San Diego, CA, USA). Significance was analyzed by independent sample t-test between two groups or one-way ANOVA among multiple groups with IBM SPSS Statistics 25 software. *p* < 0.05 was considered a significant difference.

## Data Availability

The original contributions presented in the study are included in the article/supplementary material, further inquiries can be directed to the corresponding author/s.

## Supplementary Materials

The following supporting information can be downloaded at: https://www.mdpi.com/article/10.3390/toxins16110466/s1, Table S1: Description of de novo assembly and annotation of the transcriptome. Table S2: Classification of putative polypeptides and proteins identified from transcriptome mining from *O. forcipiformis*. Table S3: Diameter of the zone of inhibition formed by GK37 against *S. aureus*, *E. coli*, *A. baumannii* and *P. aeruginosa* (mm). Table S4: The stability of GK37 in plasma within 12 h. Table S5: The prediction of hemolytic properties by AMPACTIPRED. The predicted results of GK37 are highlighted in red. Figure S1: Mass spectrum of GK37. Figure S2: High-performance liquid chromatography (HPLC) of GK37. A: 0.1% TFA in Acetonitrile. B: 0.1% TFA in H_2_O. Method: 100%H_2_O. Figure S3: Circular dichroism (CD) analysis confirmed the amphiphilic nature of GK37. TFE: Trifluoroethanol + PBS. Figure S4: Detection result of biofilm permeability of *S. aureus* by GK37. Green SYTO9-stained live cells are shown at 488 nm, and red PI-stained DNA/RNA at 594 nm. Figure S5: The RNA integrity number (RIN) result of Agilent Technologies. (a) The raw concentration of 4 RNA libraries. (b) The oligonucleotide analysis with the Agilent Electrophoresis System. Figure S6: The prediction of the hemolytic activity of GK37. (a) The predicted probability of high hemolytic activity is 71.979% of GK37 by HLPpredfuse (http://thegleelab.org/HLPpred-Fuse/index.html). (b) The predicted probability of hemolytic activity is 0.54 (0-1) of GK37 by HemoPi 2.0.
