# Supplementary material for: Peptide Toxin Diversity and a Novel Antimicrobial Peptide from the Spider Oxyopes forcipiformis
Source: Toxins (Basel). 2024 Oct 31;16(11):466. doi: 10.3390/toxins16110466 (PMC11597926; doi:10.3390/toxins16110466)
Supplement: Supplementary file 1 [file toxins-16-00466-s001.zip › toxins-3198294-supplementary.pdf]

Supplementary Materials: Peptide Toxin Diversity and a Novel Antimicrobial Peptide from the Spider *Oxyopes forcipi-formis*

Kexin Wang, James Mwangi, Kaixun Cao, Yi Wang, Jinai Gao, Min Yang, Brenda B. Michira, Qiumin Lu and Juan Li

Table S1. Description of de novo assembly and annotation of the transcriptome.

**Assembly**	
Raw reads	223.10M
Clean reads	218.28M
Number of transcripts	493,538
The average length of transcripts	999.83bp
Minimum contig	200 bp
Maximum contig	43,967bp
GC content	41.24%
**Functional annotation**	
KEGG	92,487
GO	99,318
COG	127, 368

Table S2. Classification of putative polypeptides and proteins identified from transcriptome mining from *O. forcipiformis*.

Groups	ID of sequences	ID of toxins	Reference to toxins	Similarity	Patterns of Cysteine
A	Nd20spO1:316.p2	A0A087TGE8	Defensin	45.80%	C-CXXXC-C-CXC
	Nd20spO1:38417.p2	A0A087TGE8	Defensin	45.80%	
	Nd21spO2:1277.p2	A0A087TGE8	Defensin	45.80%	
	Nd20SPT1:40431.p1	A0A087TGE8	Defensin	48.30%	
	Nd21spO2:158989.p1	A0A087TGE8	Defensin	48.30%	
	Nd21SPT2:18367.p1	A0A087TGE8	Defensin	48.30%	

	Nd20spO1:63486.p1	A0A087TGE8	Defensin	60.70%	
	Nd20spO1:25147.p1	A0A087TGE8	Defensin	68.90%	
	Nd20SPT1:10446.p2	A0A087TGE8	Defensin	68.90%	
	Nd20spO1:223.p1	A0A087T7F2	Defensin	70.00%	
	Nd20spO1:90179.p4	A0A087T7F2	Defensin	70.00%	
	Nd20SPT1:10121.p1	A0A087T7F2	Defensin	70.00%	
	Nd21spO2:378.p1	A0A087T7F2	Defensin	70.00%	
	Nd21SPT2:10943.p1	A0A087T7F2	Defensin	70.00%	
	Nd20spO1:42333.p1	A0A087T7F2	Defensin	75.00%	
B	Nd21SPT2:17771.p1	B6DCV6	U6-lycotoxin-Ls1f	37.30%	C-C-CC-CXC-CXC
	Nd20SPT1:64511.p1	B6DCV2	U6-lycotoxin-Ls1c	31.20%	
	Nd20SPT1:62212.p2	P81793	U6-ctenitoxin-Pn1a	39.40%	
	Nd20SPT1:19533.p2	A0A4Y2B7R1	U8-agatoxin-Ao1a	69.50%	
	Nd20SPT1:2277.p2	A0A087TD82	U8-agatoxin-Ao1a	68.40%	
	Nd20SPT1:43908.p2	P81793	U6-ctenitoxin-Pn1a	39.40%	
	Nd20SPT1:46068.p2	P81793	U6-ctenitoxin-Pn1a	39.40%	
	Nd20SPT1:90410.p2	P81793	U6-ctenitoxin-Pn1a	39.40%	
	Nd21SPT2:14465.p2	B6DCV2	U6-lycotoxin-Ls1c	31.20%	
	Nd21SPT2:1791.p1	A0A8X6XE39	U8-agatoxin-Ao1a	59.60%	
	Nd21SPT2:2040.p2	P81793	U6-ctenitoxin-Pn1a	39.40%	
	Nd21spO2:9845.p1	A0A4Y2B988	U8-agatoxin-Ao1a	71.40%	
	Nd21SPT2:44784.p1	A0A4Y2B988	U8-agatoxin-Ao1a	71.40%	
C	Nd20SPT1:90606.p2	P15969	Omega-agatoxin-1A	50.00%	C-CXC-CXC-CXC-C-C
	Nd20SPT1:61347.p2	P15969	Omega-agatoxin-1A	40.70%	
	Nd20SPT1:53442.p1	P15969	Omega-agatoxin-1A	39.80%	
	Nd20SPT1:18723.p1	P15969	Omega-agatoxin-1A	41.80%	
	Nd20SPT1:57739.p1	P15969	Omega-agatoxin-1A	40.80%	
D	Nd20SPT1:16201.p2	B6DD31	U2-lycotoxin-Ls1c	51.60%	C-CXCXXXCXXCXC-CXC-C-C
	Nd20SPT1:54570.p1	P15969	Omega-agatoxin-1A	48.40%	
	Nd20SPT1:79566.p1	B6DD30	U2-lycotoxin-Ls1b	45.20%	
	Nd20SPT1:77201.p1	B6DD31	U2-lycotoxin-Ls1c	46.70%	
	Nd21SPT2:16044.p1	B6DD31	U2-lycotoxin-Ls1c	46.70%	
E	Nd21spO2:58446.p1	P84093	U20-ctenitoxin-Pn1a	38.50%	C-C-CXCC-CXC-CXC-C-C

	Nd21spO2:98244.p1	P17727	Mu-ctenitoxin-Pn1a	47.10%	
	Nd21SPT2:14983.p1	P84093	U20-ctenitoxin-Pn1a	44.40%	
	Nd21SPT2:15216.p2	P84062	U5-ctenitoxin-Pk1a	42.90%	
	Nd21SPT2:14956.p1	P17727	Mu-ctenitoxin-Pn1a	40.00%	
	Nd21SPT2:23516.p2	P84093	U20-ctenitoxin-Pn1a	40.90%	
	Nd21SPT2:60757.p1	P84093	U20-ctenitoxin-Pn1a	38.50%	
	Nd21SPT2:43609.p2	P17727	Mu-ctenitoxin-Pn1a	47.10%	
	Nd21SPT2:54081.p3	P84093	U20-ctenitoxin-Pn1a	40.90%	
	Nd21SPT2:60757.p1	P84093	U20-ctenitoxin-Pn1a	38.50%	
	Nd21SPT2:60757.p1	P84093	U20-ctenitoxin-Pn1a	38.50%	
	Nd20SPT1:2955.p1	P17727	Mu-ctenitoxin-Pn1a	37.00%	
	Nd20SPT1:43405.p1	Q5Y4U3	U9-agatoxin-Ao1a	54.70%	
	Nd21SPT2:15761.p1	P84093	U20-ctenitoxin-Pn1a	43.10%	
	Nd21SPT2:2128.p2	P17727	Mu-ctenitoxin-Pn1a	34.70%	
	Nd21SPT2:240.p1	P84093	U20-ctenitoxin-Pn1a	41.40%	
	Nd21SPT2:4631.p1	P84093	U20-ctenitoxin-Pn1a	41.40%	
	Nd21SPT2:587.p1	P17727	Mu-ctenitoxin-Pn1a	37.60%	
	Nd21SPT2:11554.p1	P84093	U20-ctenitoxin-Pn1a	44.60%	
F	Nd21SPT2:1646.p2	P17727	Mu-ctenitoxin-Pn1a	38.50%	C-C-CXCC-CXC-CXC-C-C-C
	Nd21SPT2:1748.p2	P17727	Mu-ctenitoxin-Pn1a	38.50%	
	Nd20SPT1:101.p1	Q5Y4U3	U9-agatoxin-Ao1a	50.60%	
	Nd20SPT1:25571.p1	Q5Y4U3	U9-agatoxin-Ao1a	42.50%	
	Nd20SPT1:3238.p1	Q5Y4U3	U9-agatoxin-Ao1a	54.00%	
	Nd20SPT1:892.p1	Q5Y4U3	U9-agatoxin-Ao1a	42.50%	
	Nd21SPT2:1908.p1	Q5Y4U3	U9-agatoxin-Ao1a	45.10%	
	Nd21SPT2:1937.p1	Q5Y4U3	U9-agatoxin-Ao1a	50.60%	

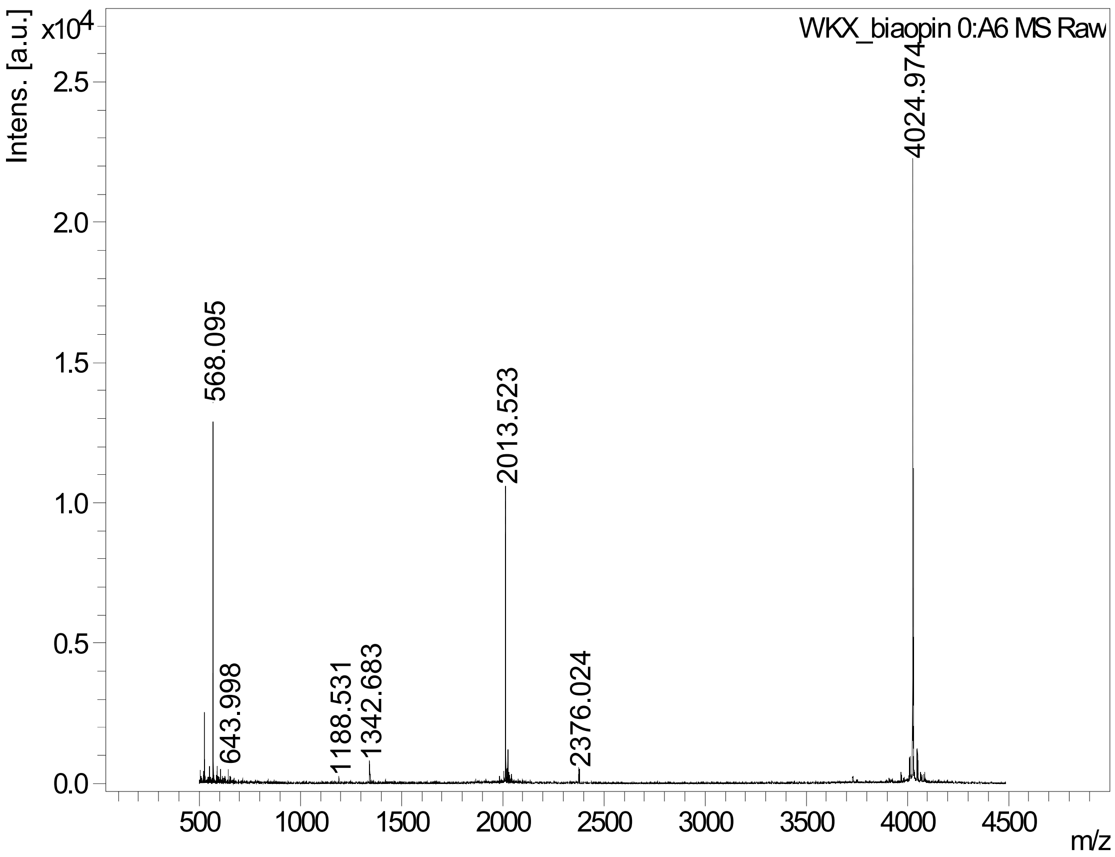

Figure S1. Mass spectrum of GK37.

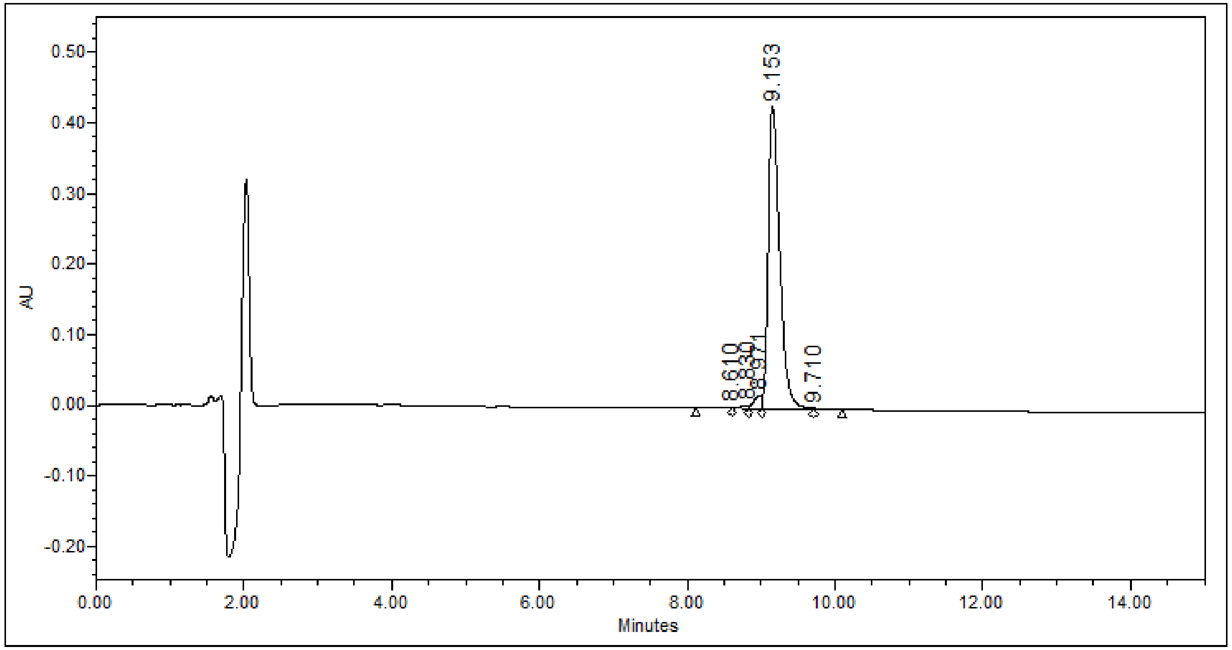

	RT	Area	% Area	Height
1	8.610	25688	0.53	1490
2	8.830	33938	0.70	4241
3	8.971	134588	2.77	18508
4	9.153	4650976	95.58	427776
5	9.710	20914	0.43	1474

Figure S2. High Performance Liquid Chromatography (HPLC) of GK37. A: 0.1% TFA in Acetonitrile. B: 0.1% TFA in H2O. Method: :100%H2O.

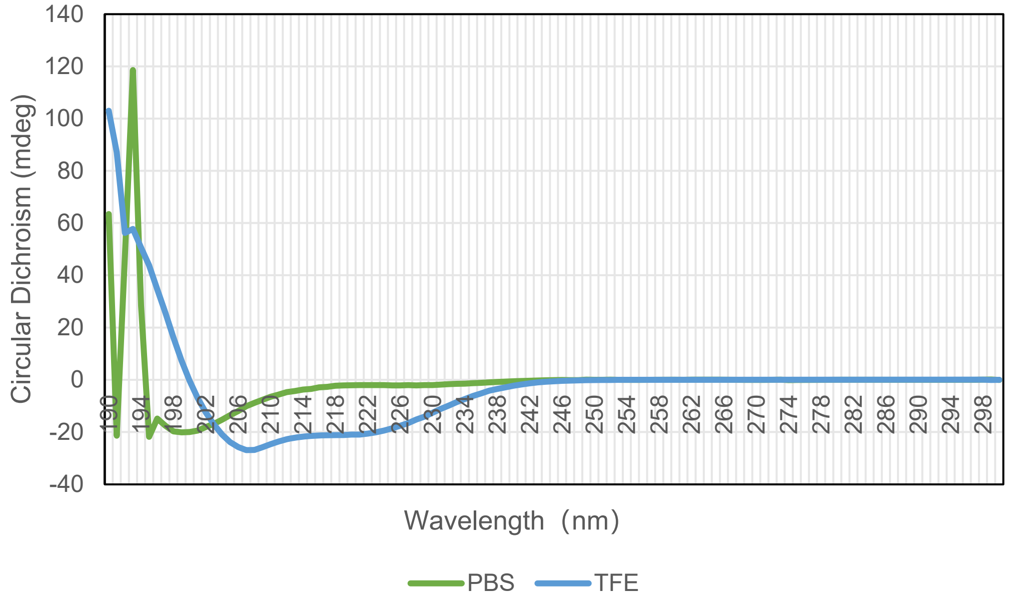

Figure S3. Circular dichroism (CD) analysis confirmed the amphiphilic nature of GK37, TFE: Trifluoroethanol + PBS.

Table S3. Diameter of the zone of inhibition formed by GK37 against S. aureus, E.coli, A.baumannii and P.aeruginosa (mm).

Bacteria strain	Inhibition Zone Diameter (mm)		
	GK37	Vancomycin	Colistin
S. aureus (ATCC6538)	14.12	7	/
E. coli (ATCC8739)	9.59	/	18.94
A. baumannii (ATCC19606)	11.99	/	/
P. aeruginosa (ATCC27853)	7.58	/	19.5

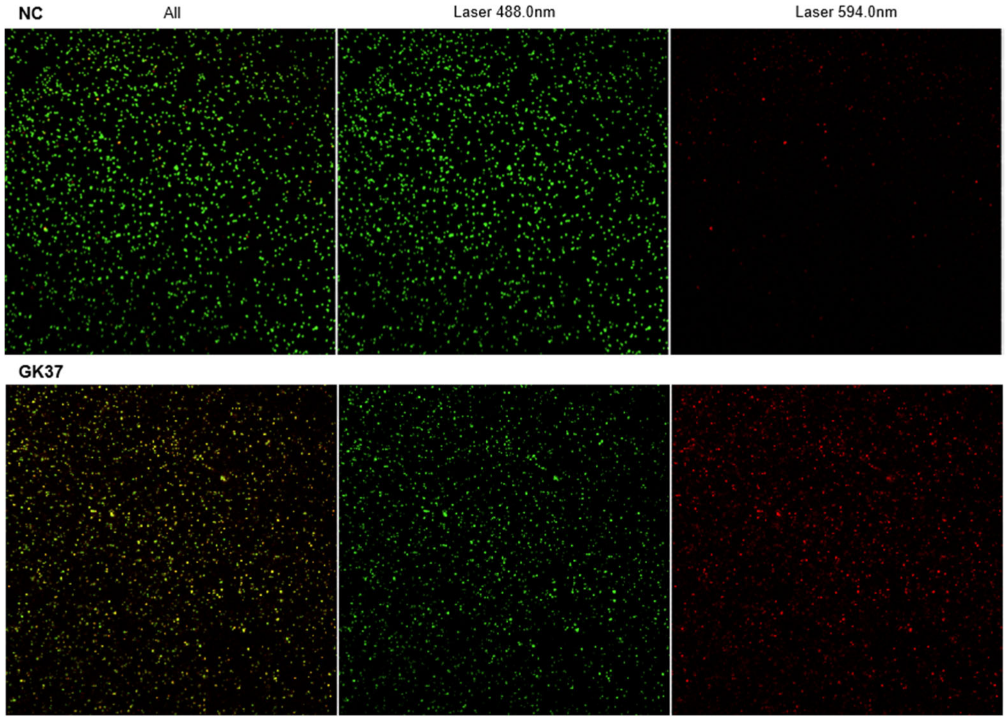

Figure S4. Detection result of biofilm permeability of S.aureus by GK37. Green SYTO9-stained live cells are shown at 488 nm, red PI-stained DNA/RNA at 594 nm.

Table S4. The stability of GK37 in plasma within 12 h.

**Time（h）**	**0**	**1**	**2**	**4**	**6**	**8**	**10**	**12**
MIC （μg/mL）	6.25	6.25	6.25	6.25	6.25	6.25	6.25	6.25

Table S5. The prediction of haemolyticproperties by AMPACTIPRED.The predicted result of GK37 are highlighted in red.

SeqID	Predic-tionclass	Prediction probability	Sequence
Nd21SPT2 _91688.p2	AMP	0.9996	GKFSVFSKMLKSIAKFFKGVGKARKAFKDASDLDKNQ
Nd21SPT2: 100372.p1	AMP	0.9995	EKSSAEVQGTPMSKCRIMKNLMIMCLGFLFLFCAFQSLASLQTTINQQGSLSQSLI- YACLAMSSLLLPKLLISKLGCKATLVMSMATYMPFMASNYYNDFLTQILTSMLL GLGGGTLWASACTYMNEISVLYATHLADSVDVMTTSFFGMFFMMFQNS- QIWGNLATFYIL- SPTDEEVLKSMLLSASNVTVAGEIFNSSTVACGANFCGGVSSSETQISETKRFILMS AYLGFTALSAVMVAVFLDPLQSKDLEKDSTMLSSVTATIKHLRK- PDQLLLMPMTMFCGFQQAFVLSEFSQAYMACAWGVHNVSLVFMFFGAVNSVA SFLVGSIAKYIPSLCVLLTGAMGNTFACIILFLWDPDGSIIYYFIVIGLWGLSDAI- WQTEMNSMYGMLFSSDEEAAYGNLRLWEAVGFFLPYFFNTLLCMSSKLYFIIALL TVSITGAAIVEVKTSFKKKS
Nd21SPT2: 100373.p1	AMP	0.9995	EKSSAEVQGTPMSKCRIMKNLIIMCLGFLFLFCAFQSLASLQTTINQQGSLSQSLI- YACLAMSSLLLPKLLISKLGCKATLVISMATYMPFMASNYYNDFLTQMLTSMLL GLGGGTLWASACTYMNEISVLYATHLSDSVDVMTTSFFGMFFMMFQNS- QIWGNLATFYIL- SPTDEEVLKSTLLSASNVTVAGEIFNSSTVACGANFCGGVSSSETQISETKRFILMS AYLGFTALSAVMVAVFLDPLQSKDLEKDSTMLSSVTATIKHLRK- PDQLLLMPMTMFCGFQQAFVLSEFSQAYMACAWGVHNVSLVFMFFGAVNSVA SFLVGSIAKYIPSLCVLLTGAMGNTFACIILFLWDPDGSIIYYFIVIGLWGLSDAI- WQTEMNSMYGMLFSSDEEAAYGNLRLWEAVGFFLPYFFNTLLCMSSKLYFIIALL TVSITGAAIVEVKTSFKKKS
Nd21SPT2: 6665.p1	AMP	0.9999	RNLLIISFAYFLYFTGFWSLANLQSTMNAEQGLGPNSQAVMYFCSMLSCFF- PQLMIEKIG- SKTTFIMALLVSCPFMAANIHLRWDIMMASSVLYGFASGPLNAAMLLYLDEMAT SYKSAVSESKENVKAFFFGVNIFFAEITQVLGSAISYFVLD- GDAEAHLASNASQRCDCGVDFYPGPESDNNTNLVPPSENESMTLTGIYLMLAVS SVMFALFLDPLKNDVKDISGCQTVSKTFFGAVNLLKNPHQLLLIPLSMFLGIEGA- FYA- NEFTEAFIACSWGVHHVGFVTICFGVCGALMAVLVGPMVKCVSQMSVIVFAALI

			NVVTCAMLFVWEPSPDEPVIYFLFAGVWGMTDAIWWSQMAALYGLMFPSDRE-<br>AAFSNFFFWSFLGYFLSYSYANYFSVAIKINIMLVFLVVGITLYVIGQVKIKCC
Nd21SPT2:34035.p1	Non-AMP	0.004	MSKCRIMKNLMIMGLGFFLLFCAFKSLESLQTT-<br>MNHQGSVSESLIYASFAVSSLLLPKLLISKLGCKATIVMSMATYIPFMASNYYNGF<br>LTLLPTSILSGLGGATLWSSACTYLNEISFLYSGHVSDSVDVITTSFFGMFF-<br>MMFQNS-<br>QIWGSLASFYILKPSGDEIFEAKLLGSSNATLVGESKNLSYVPCGANFCGGVSSSET<br>QITEAKRFTIMSVYLSFSVLSTLMVGIFLDPLPKSDLDEDSSTFSNLTATVKHMRK-<br>PDQLLLIPLTIFNGFEQAFVLSEFSQVMHWNSHCLAL
Nd21SPT2:42340.p1	Non-AMP	0.0298	LSDEECLKERKWWCFLLSSIITFLVGLFIILIWRAFAFICCRKATGDTQAAT-<br>KTKVPDSKSGGGNNSEVTEIGFMTEAKDWAGELISGQTTTGSMLVVLVFLLSMA<br>SLIIYFMDASKDKVETCHPWSENTTQQIDLALNMFFMVYFFMRFIAASDKLWFM-<br>LELYSFVDYFTIPPSFISIYLGSTWIGLRFLRALRLMSVPDILQYLNVLKTSSSIRLAQ<br>LVSIVMSVWLTAAGIIHLLENSGDPLEFSNPNEMTYWQCVYFLMVTMSTVGYG-<br>DIYCQTTLG-<br>SAFMVLFILVGLAVFASWIPEITELIGHPSKYGGVLSKERGKSHIVVCGHINYDSVS<br>YFLKDFLHEDSEDVDVEVVFLHSKPPDLELEGLFKSHFT-<br>TVEFFQGTVMNPVDLQRVKVHQADACLVLANKYCQDPDAEDAANIMSVISIKN<br>FSDDISVIIQLMQYHNKAYLLNIPSWNWKNGDDVICLAELKLGFIAQSCLAPGF-<br>STMVANLFAMRSYKTSPDTPAWQNDYLCGTGMEMYTETLSTSFVGMSFPQAAE<br>LCFVKLKLLLLAIEVTNEEGTDTKISINPKSQTKLSPDTQGFFMAQSADEV-<br>KSVWYYCKNCHEDVKDEKQIKKCKCKIPPSSSVVLPRNGNRQKSPGKVNSMSSIS<br>SASKGSHGSPTGRNSSGSVTESPFGIMAEDQGKDFD-<br>FESTEMKYDSTGMFHWCPARSMEDCMLDRNQAAMTVLNGHVVLCLFADKDSP<br>LIGLRNLVMPLSASNFHYHELKHVVIVGNVDYL-<br>GSEWKMLQNLPKMSILSGSPLSRADLRAVNINLCDMCVMLSAKIPSSEDPTLAD<br>KEAILASLNIKAMTFDDTIGVLTHHETGREGMSPLGSPIVLQSSGSVYGTNVP-<br>LITELVNDTNVQFLDQDDDDDPDTELYLTQPFACGTAFAVSVLDSLMSTTYFNA<br>NALTLIRSLITGGATPELELILAEGAGLSGGYSTPESLNNRDSCRVGQMSLSDG-<br>PLA-<br>SYGEGGKYGDVFVKALKNYGMLCIGLYSYSDTSSSFEASTKRYVITNPPAEFPLMP<br>SDKVFVLMQFDPGLEYQPNRGDRDDNS

Nd21spO2:<br>1846.p1	AMP	0.9498	HYADEQDMCHSMENVPTKSVGEPKLKNPPMFFSKLSMMKNAFILCLVFFMT-<br>FIAYDCLS-<br>MLQSTMNHDDGIGVICQAITYACFCISSLLLPKYVMKKLGCKTTLVLSMCLYMPY<br>IASNLYPHWAFMVPAAVMNGLVASLLWGSQSIYLNEIAKLYVSQSLASKNN-<br>KLFCMGSNNDSNVMSTMKITPKTQSFSNEQVCEKEKEVTPSTSSSENPLTDRYYE<br>CFPTDCNTLNNKNDAKFLPSSAKNVETQIHVKNMVAKFFGVFGMAYLSTH-<br>MWSNLM-<br>SYFILMDDSQHNNATFNSSCVCGAQYCNVESVCFMNNLQEPSLHNRYLLTGMC<br>MALGVVSILILALCLDPLNDRTQKMKFSFDLLFATCKHAKKKNQLFLT-<br>PLSLYVG-<br>MIQGFYSGDFNMSYVGCAWGTYHVGLVSVCYGAMCGTSSSLSGCLVTKIGSVPV<br>LTFAAVVNAAAIILLLSWKPTAENTEMFFVTAGLWGTSVGILWSQLKAMYG-<br>FIFKKEEEAAFGAYHLWYSLGFSMSFAYSNHLCTYIKIYMLLSVLILGTIGYLYVEV
Nd21SPT2:<br>68553.p1	AMP	0.9995	CSGQSNCECCGDDTYCWCLWGMSIGCSCLKNYSQSLCLEKYDCMNRHLWNNK
Nd21SPT2:<br>72234.p2	AMP	1	CSNIGEECWYDIKPCCNSVPCKCNLLLTSCKCK
Nd21SPT2:<br>67629.p2	AMP	0.9991	GFPCDKNKTECQCCGESTHCLCGWGLPFIGECKCHDGTKDICISKGNCSNREEW
Nd21SPT2:<br>3611.p2	AMP	1	CTGYCSFCNCKMLLFSADLADGCTPLSQWCDPYIESPCQCCGDY-<br>SYCWCFWGLKWLRICKCSAGSKKICEEKLSCPNRWSW
Nd21SPT2<br>_93743.p1	AMP	0.9996	GKFSVFSKMLKSIAKFFKGVGKARKAFKDASDLDKNQ
Nd20SPT1:<br>79745.p1/G<br>K37	AMP	0.9996	GKFSIFGKILSSIAKVFKGVGKVRKSFQNASDLDK
Nd20spO1:<br>63486.p1	AMP	0.9999	ATSSNENTCPSNWSACDNLCKSSGRIAGYCAGIWSHSCTCVKRGLS-<br>MTSDIVPPWG
Nd21SPT2:<br>71700.p1	Non-AMP	0.3481	FYTVACFWAFSDAIWKTQLSALYGVLFSSDQEAAFANYSMWEAVGYL-<br>IAFGCSNVLCMSYKIYMQIAFLLVGMAGYMMVELSIFRSKK
Nd21SPT2:<br>68826.p1	AMP	0.9996	EFKYALLANNCLCPGTSTLVTLLLHTSSGQEGQMS-<br>GEEWHSLYGKCSGNEIYHIRLGDSS-<br>FFGEYEGKPFNFASFHSHSKYGVALVGVKTEMGGSWPVLLNPGPNYVLKAS

Probabilities > 0.5 = Predicted AMP

(a)

Library Name	Library type	Nucleic acid number	Concentration (ng/μL)	Volume (μL)	Amount (μg)	Integrity value	Testing reult	Note	Sample volume (μL)
20SP-T-1	ECT	FZTR220131080-1A	148.000	35.00	5.18000	8.50	Pass	Smooth baseline	/
21SP-T-2	ECT	FZTR220131081-1A	152.000	35.00	5.32000	7.10	Pass	Smooth baseline	/
20SP-O-1	ECT	FZTR220131074-1A	1919.000	32.00	61.40800	6.90	Pass	Smooth baseline	0.10
21SP-O-2	ECT	FZTR220131075-1A	342.000	32.00	10.94400	6.00	Pass	Smooth baseline	0.50

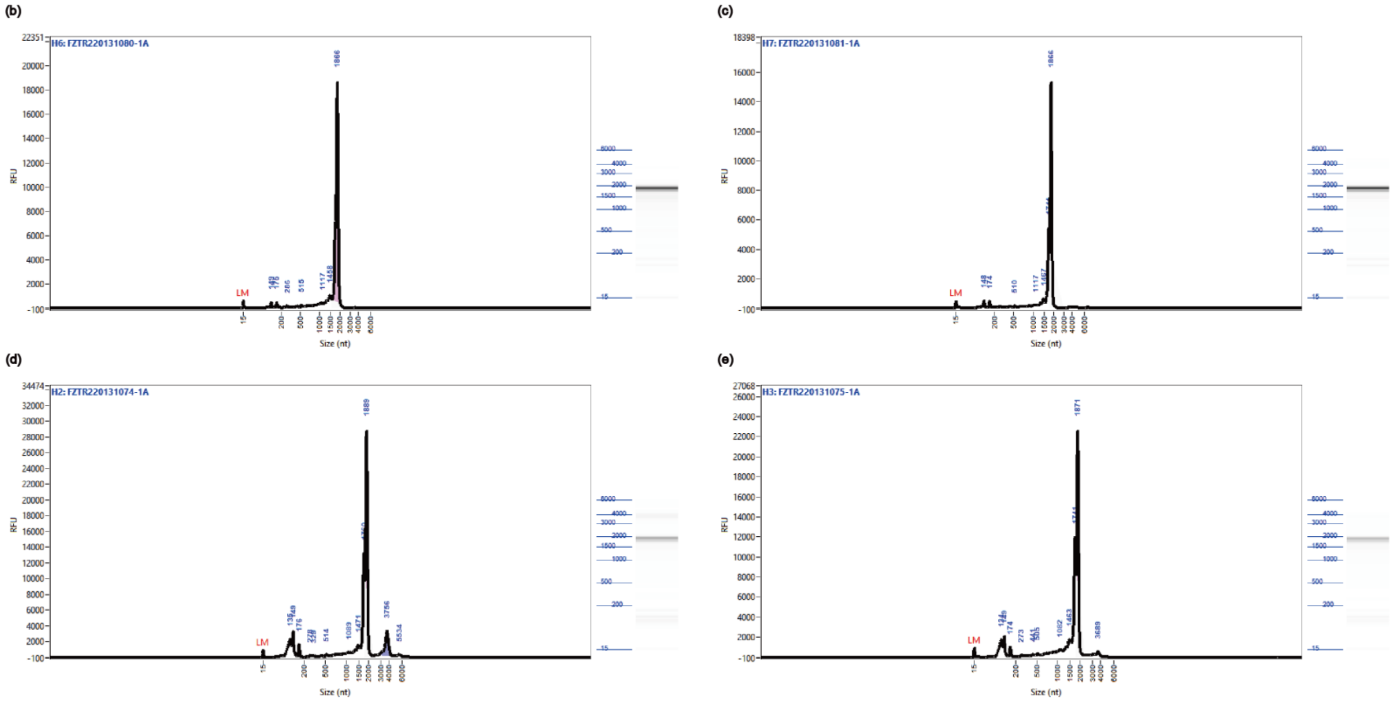

Figure S5. The RNA integrity number (RIN) result of Agilent Technologie. (a) The raw concentration of 4 RNA librarys. (b) The oligonucleotide analysis with the Ag-ilent Electrophoresis System.

(a)

High or low represent high or low haemolytic activity

S.NO	FASTA ID	High or Low	Predicted probability of High
1	GK37	High	0.719791666667

(b)

PROB score is the normaliazed SVM score and ranges between 0 and 1, i.e. 1 very likely to the hemolytic, 0 very unlikely to be hemolytic.

Query Peptides

Peptide ID	peptide Sequence	PROB Score	Hydrophodicity	Hydropathivity	Hydrophilicity	Charge	Mol wt
GK37	GKFSIFGKILSSIAKVFKGVGKVRKSFQNASDLDK	0.54	-0.15	-0.08	0.21	6.00	3801.97

1/1 50

Figure S6. The predication of the haemolytic activity of GK37. (a) The predicted probability of high haemolytic activity is 71.979% of GK37 by HLPpredfuse (http://thegleelab.org/HLPpred-Fuse/index.html). (b) The predicted probability of haemolytic activity is 0.54 (0-1) of GK37 by HemoPi (https://webs.iiitd.edu.in/raghava/hemopi/batch.php).
